# Tensile and Shear Creep Behavior of Structural Adhesives: Experiments and Modeling

**DOI:** 10.1007/s10443-023-10184-y

**Published:** 2023-12-30

**Authors:** Gilda Daissè, Bilen Emek Abali, Roman Wan-Wendner

**Affiliations:** 1 Christian Doppler Laboratory, University of Natural Resources and Life Sciences Vienna, Peter-Jordanstr. 82, Vienna, 1190 Austria; 2 Division of Applied Mechanics, Uppsala University, Box 35, Uppsala, 751 03 Sweden; 3 Department of Structural Engineering and Building Materials, Ghent University, Tech Lane Ghent Science Park - Campus A, Technologiepark-Zwijnaarde 60, Ghent, 9052 Belgium; 4 Department of Material Sciences and Process Engineering, University of Natural Resources and Life Sciences Vienna, Peter-Jordanstr. 82, Vienna, 1190 Austria

**Keywords:** Creep, Structural adhesive, Curing, Continuous spectrum, Thermoset polymers

## Abstract

Structural adhesives characterized a turning point in the post-connection of structural elements due to their excellent performances and ability to transfer stress without losing their integrity. These materials are typically particle-reinforced composites made by a thermoset polymer matrix and fillers. During the in-situ application of this material, the thermal activation of the polymer is typically not possible, leading to an undefined degree of cure and therefore to a variation of the mechanical performance over time. This altering means that after applying a sustained load on a bonded anchor system installed at regular temperature, the adhesive changes material properties. Ample studies convince that the progressive increase of the degree of cure of the thermosetting polymer leads to higher strength and stiffness. However, limited studies have been dedicated to the post-curing effects on the long-term behavior. The main goal of this work is to investigate the tensile and shear creep behavior of two commercially available structural adhesives and the influence of curing conditions on their long-term performances. An extensive experimental campaign comprising short and long-term characterizations has been carried out on specimens subjected to three different curing and post-curing protocols, with the scope of imitating relevant in-situ conditions. The results demonstrate that structural adhesives cured at higher temperatures are less subjected to creep deformations. As a material equation, the generalized Kelvin model is utilized to fit the tensile and shear creep data, and two continuous creep spectra have been selected to represent the creep behavior and facilitate extrapolations to the long-term behavior.

## Introduction

In the last decades, structural adhesives have been widely used in construction and civil engineering, particularly in connection with strengthening techniques and in the post-connection of structural elements. These materials are typically particle-reinforced thermoset polymers that harden rapidly at room temperature via a chemical process called curing and allow a fast application on the construction site. Due to their ability to redistribute the stress, the possibility of joining different materials, and the reduction of the overall weight and manufacturing costs [1], these materials are often used in post-installed anchor systems, so called bonded anchor systems. Anchorage failure may lead to severe consequences, and for this reason, approval tests for fastening systems [2–4] must cover short and long-term responses [5, 6]. Mainly motivated by investigating the unfortunate causes of two accidents, where ceiling panels of tunnels have been collapsed [7–9], the knowledge of the long-term deformability of structural adhesives became essential. In recent decades, numerous investigations pertaining to the phenomenon of creep in bonded anchors have been conducted [10–15]. Muciaccia et al. [10] examined the creep behavior of bonded anchors under high sustained tensile loads, highlighting the inadequacy of current assessment criteria. The study from Mahadik and Hofmann [11] explores the creep behavior of adhesive anchors under sustained loads and proposes an alternative evaluation method based on time-to-failure and displacement criteria. The investigation of Djeumen et al. [13] focused on the creep behavior of adhesively bonded fasteners under tension and shear loads, revealing non-uniform stress distributions and the need for load-dependent parameters in the creep model.

During the pull-out of a bonded anchor system, the adhesive layer is subjected to a combination of tensile and shear forces. Therefore, for computing the long-term performance of these systems precisely, tensile and shear creep behavior need to be incorporated in simulations.

In the existing literature, there are various viewpoints regarding the suitable approach for testing structural adhesives. When the primary objective is to evaluate the adhesive’s performance in joining different materials, researchers often focus on examining the adhesive joint interphase [16–19]. However, when the main goal is to determine the intrinsic material properties of the adhesive itself, tests conducted on bulk adhesive specimens are commonly used [20]. As this study specifically aims to assess material performance, the selected structural adhesives will be evaluated as bulk specimens. This work investigates the creep behavior of two commercially available structural adhesives, one epoxy-based and the other vinyl ester-based, subjected to tensile and shear loading. Short creep tests were performed by uniaxial tensile loading, where so-called "dog-bone" specimens were employed according with the DIN EN ISO 527-2: 2016-06 standard [21]. Additional short creep tests were conducted in shear by adopting the Iosipescu configuration [22]. The experimental campaign also includes long-term gravity loaded creep tests performed in tension, where deformations up to 2 years were measured and used as valuable data for model validation.

Long-term creep tests were performed on fully cured specimens in order to exclude a possible contribution of post-curing effects (also called physical aging) on the adhesive. A thermoset polymer is considered as fully cured when the polymerization reaction is completed, and this state is achieved by post-curing the specimens at high temperatures [23–28]. On the construction site, thermal treatments are rarely carried out on site as it requires specific equipment, and the degree of cure of the adhesive is unknown. Yet we know that the mechanical properties of structural adhesives are highly dependent on the degree of cure [25, 29–39] which in turn depends on curing temperature and time. Therefore, the main scope of the presented work is to study the creep behavior of these materials for different curing states derived from relevant in-situ conditions. Short creep tests were performed on samples with three different degrees of cure. The time range used for these tests is considered to be short enough to minimize post-curing effects and obtain relevant information on the influence of curing on long-term properties.

When a material is subjected to a sustained load, its creep behavior can be categorized into three distinct stages: i) primary creep - is the initial transitional phase where the material experiences a rapid increase in creep strain, ii) secondary creep - when the material reaches a steady state where the creep strain increases linearly over time, and iii) tertiary creep - is the final stage of creep, which can ultimately lead to failure. The latter stage is crucial for assessing the lifespan and durability of adhesively bonded joints, and several investigations have been conducted to better understand it [40–42].

In the literature, different models are proposed to predict the secondary creep behavior of thermosets [43–50]. The three main rheological models are the Maxwell, Kelvin, and Burger models; they incorporate viscous behavior into the stress-strain relation often visualized by means of Hookean springs and Newtonian dashpots in analogy of force-displacement relations. Modifications of the Burger model are proposed by Majda and Skrodzewicz [46], and Costa and Barros [47] for epoxy adhesives. Silva et al. [48] proposed a framework based on the generalized Kelvin model capable of simulating the creep behavior of epoxy for different loading ages. Other authors [51, 52] have made modifications to rheological models in order to incorporate the non-linear stress dependence of creep behavior. This stress dependency is generally observed at high stress levels. However, it has been demonstrated that for low stress levels, the creep behavior of thermoset materials can be considered linear [48, 53].

While these analytical models accurately replicate the creep behavior of epoxies, their validity is limited to the time interval of the underlying experiment, in other words, they fail to extrapolate a material’s behavior and predict substantially longer time periods than tested. Additionally, the inverse determination of spring and dashpot properties from observed creep deformation histories is an often ill-posed task.

In order to predict the long-term properties of polymers, the time-temperature superposition principle is typically employed [1, 54–57]. This method involves constructing a master creep curve from a collection of curves obtained from short creep tests conducted at different temperatures. The creation of this curve requires evaluating various material properties and determining the shift factor [58]. However, this methodology is only applicable for fully cured specimens such that the shift factor remains constant during the experiment. We aim at modeling incompletely cured systems. Therefore an accelerated test via temperature increase alters the degree of cure and is unsuitable for the intended research.

This paper introduces two key novelties. Firstly, the influence of curing and post-curing treatments on the creep behavior of two structural adhesives, in both tension and shear, is explored. This analysis presents new insights into the behavior of epoxy and vinyl ester-based materials. Secondly, the generalized Kelvin model, enhanced with two continuous retardation spectra, is utilized to simulate this creep behavior. The Kelvin model outputs a discrete spectrum, with accuracy dependent on the number of elements in the chain and the selection of retardation times [59]. To address this, the implementation of two continuous spectra in the model is proposed, offering a more robust method for parameter identification and time extrapolation. The effectiveness of using a power law function as a continuous retardation spectrum for accurate time extrapolation of data is demonstrated. This spectrum is initially fitted using data from 24-hour tests and subsequently employed to predict the creep curves over periods of 2 and 50 years. The 2-year prediction has been validated with experimental data.

## Materials

Two commercially available structural adhesives have been studied. Both materials are particle-reinforced thermosetting polymers with a relatively high content of inorganic fillers. The two materials exhibit different compositions: one is an epoxy-based resin (abbreviated EP) composed mainly by Bisphenol-A-diglycidylether, Bisphenol-F-diglycidylether, and m-Xylylenediamine; while the second product is a vinyl ester-based (abbreviated VE) product mainly composed of 1,4 Butanediol dimethacrylate and 2-Hydroxypropyl methacrylate. Both materials contain quartz particles but are characterized by different microstructures. The VE contains both small and large fillers ($\sim$20-200 $\upmu$m) with a rounded shape, and the failure is usually localized between the particles and the matrix. The EP includes only small fillers ($\sim$20 $\upmu$m), and the crack propagation may also pass through the fillers. Further information about the composition of the two products can be found in Table 1 and in several other studies [29–33, 54, 55, 60].

**Table 1:** Matrix, filler type, and content of the two adhesives, EP and VE

**Product**	**Matrix**	**Filler type**	**Vol. of fillers**	**Dimension of fillers**
EP	Epoxy	Quartz	19%	$\sim$20 $\upmu$m
VE	Vinyl ester	Quartz	46%	$\sim$20–200 $\upmu$m

The thermoset polymer matrices characterize the two materials’ mechanical responses. Their characteristics are strongly determined by the degree of cure that is mainly set by the curing temperature [61]. Daissé et al. [34] showed how the curing and post-curing treatments largely influence the strength and the stiffness; Singer et al. [29] demonstrated that time and temperature are critical parameters in determining the final material properties, and Siedlaczek et al. [31] underlined the effect of hydrolytic aging on the materials’ performance.

In the aerospace and automotive industry, the influence of the matrix on the mechanical properties is minimized by acquiring the fully cured state. Yet in the civil engineering field, the in-situ conditions vary substantially and thermal activation of adhesives is typically impossible. For this reason, this study investigates the creep behavior of the two products under different curing and post-curing protocols, representing practically relevant in-situ conditions. All the samples are cured inside silicon molds at 23 ^∘^C for 2.5 h (VE) or 24 h (EP) and then post-cured at different temperatures inside an oven. Table 2 reports the curing and post-curing protocols to which the specimens were subjected to acquire three different curing states. At the end of the thermal process, the degrees of cure of the epoxy-based material was determined by means of differential scanning calorimetry (DSC) measurements, while the change of state in vinyl ester was assessed by gravimetric measurements. The latter ones have been conducted to assess the influence of curing and post-curing, where DSC measurements could not be performed successfully. Specifically, in the case of vinyl ester samples, several attempts were made to perform DSC measurements. However, due to the material composition and the rapid reaction characteristics of vinyl ester, the DSC measurements did not provide conclusive results. Therefore, alternative testing methods were employed to obtain relevant information concerning the curing state of the material.

**Table 2:** Curing and post-curing protocols for generating three EP and three VE test examples and the correspondent degree of cure and mass change [34]

**Product**	**Protocol**	**Curing**	**Post-curing**	**Degree of cure**, $\phi$ [-]	**Mass change**, $M_t$ [%]
EP	EP1	23 ^∘^C/24 h	23 ^∘^C/24 h	0.84 ± 0.02	-
	EP2	23 ^∘^C/24 h	43 ^∘^C/24 h	0.95 ± 0.01	-
	EP3	23 ^∘^C/24 h	110 ^∘^C/24 h	0.975 ± 0.005	-
VE	VE1	23 ^∘^C/2.5h	23 ^∘^C/24 h	-	$-$0.512 ± 6.7
	VE2	23 ^∘^C/2.5h	72 ^∘^C/24 h	-	$-$2.596 ± 4.1
	VE3	23 ^∘^C/2.5h	110 ^∘^C/24 h	-	$-$4.965 ± 2.2

The reported degree of cure $\phi$ have been calculated as the ratio between the total heat released during the test (*H*) and the total reaction enthalpy ($\Delta H_R$):

1 $$\begin{aligned} \phi = \frac{H}{\Delta H_R} \end{aligned}$$

While the mass change $M_t$ is given by the difference between the weight of the sample after and before the thermal treatment:

2 $$\begin{aligned} M_{t} = \frac{W_{t}-W_{0}}{W_{0}} \times 100\% \end{aligned}$$

Further details about the curing states are provided in [34].

## Experimental Campaign

The purpose of this study is to analyze the long-term behavior of two structural adhesives cured at different temperatures. The experimental campaign includes quasi-static tests, loaded directly to failure, and creeps tests. Both types of tests are available in tension and shear. The complete test matrix with the corresponding number of tested specimens is reported in Table 3.

**Table 3:** Test matrix providing the number of tested specimens for different protocols as indicated in Table 2

**Product**	**Protocol**	**Tension**		**Shear**	
		**Quasi-static**	**Creep**	**Quasi-static**	**Creep**
EP	EP1	3	5 + 4^a^	3	3
	EP2	3	5	3	3
	EP3	3	5	3	3
VE	VE1	3	3	3	3
	VE2	3	3	3	1
	VE3	3	3	3	1

^a^Long-term creep tests

### Quasi-Static Tests

**Fig. 1:**
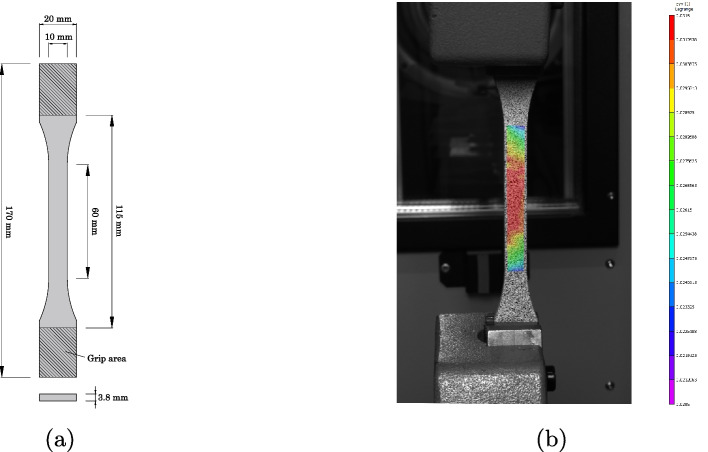
Tensile test specimen: (**a**) Geometry of the specimen and (**b**) DIC image

Tensile tests were performed on the so-called “dog-bone” specimens, geometry 1B type of the DIN EN ISO 527-2: 2016-06 standard [21]. The specimens were obtained by injecting the product into a closed silicon mold. The two structural adhesives are available in cartridges in which the hardener and the resin are separately stored. For mixing the two components adequately with the same ratio, the material is pushed through a screw-on static mixer tip with a press. At the end of the curing time (reported in Table 2), the specimens were post-cured in an electronically controlled drying oven (Memmert, UE 500). After the thermal treatment, the samples were ground at 300 rpm on a rotating plate of a grinding machine (Struers, Planopol-3) using a resin-bonded diamond grinding disc (Struers, MD-Piano series). The thickness of each specimen was assessed with a caliper (precision 0.01 mm), and the cross-sectional area in the central region was recorded. The geometry and the dimensions of the dog bones are reported in Fig. 1a. In order to minimize the aging effect, the specimens were sealed and stored in a fridge with a temperature of 6 ^∘^C until the testing day. The time span between the casting and the testing was $4-6$ days.

**Fig. 2:**
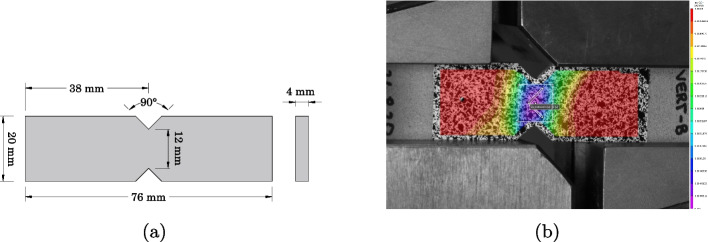
Shear test specimen: (**a**) Geometry of the specimen and (**b**) DIC image

The tensile tests were carried out using a ZwickRoell Z010 10 kN machine according to the standard DIN EN ISO 527-2: 2016-06. The tests were performed in stroke control with a loading rate of 0.5 mm/min. The strain was monitored using a DIC (Digital Image Correlation) stereo system (Correlated Solutions), consisting of two Prosilica GT3400 9.2 Megapixel CCD cameras with 25 mm lenses with an optical resolution on the object of 8.79 pixels/mm. In order to obtain an optimal pattern, the specimens were painted with a white color and then sprayed with a black color. The resulting speckles had a size of approximately $5\times 5$ pixels. At the end of the tests, the fracture surfaces of the specimens were visually inspected to detect the presence of bubbles or imperfections.

Shear tests were performed on rectangular V-notched samples, schematically shown in Fig. 2a. The samples were cast with the two notches in silicon molds, following the same procedure as described for the tensile specimens. The cross-sectional area between the two notches was measured with a caliper (precision 0.01 mm) and recorded in the testing protocol. Iosipescu shear tests according to ASTM standard D5379/D5379M-98 [22] were performed using a ZwickRoell Z010 10 kN machine. The Iosipescu shear set-up consists of two parts: one part fixes half of the specimen by applying compression, and the second applies the vertical load. The shear angle $\gamma _{xy}$ was monitored by means of a Digital Image Correlation (DIC) system as demonstrated in Fig. 2b and described in detail in Section 3.1. The tests were performed by controlling the displacement at a constant velocity of 0.5 mm/min for the epoxy and 0.2 mm/min for the vinyl ester in order to reach the failure load in a about 1 and 3 min, as indicated in the EAD 330499-01-0601.

### Creep Tests

Tensile and shear creep tests were performed using the same specimens described for the quasi-static tests. The purpose of these tests is to examine the secondary creep stage of the two materials. Therefore, low load values were applied to remain in the linear elastic range and avoid damage. The force was applied as load to the specimen in displacement control with a speed equivalent to the quasi-static loading rate until the target load was reached. After reaching 20% of the maximum strength, the loading was kept constant. We compile all values in Table 4.

**Table 4:** Ultimate strength and creep loads in tension and shear

**Product**	**Tension**		**Shear**	
	$\sigma _\text {ult}$ [MPa]	$\sigma _\text {creep}$ [MPa]	$\tau _\text {ult}$ [MPa]	$\tau _\text {creep}$ [MPa]
EP1	45.5 ± 6.8%	9.1	45.2 ± 3.1%	9.0
EP2	66.1 ± 2.0%	13.2	50.2 ± 5.2%	10.0
EP3	76.8 ± 3.7%	15.4	55.4 ± 2.3%	11.1
VE1	12.5 ± 3.7%	2.5	14.4 ± 3.5%	2.9
VE2	15.6 ± 4.0%	3.1	17.8 ± 3.3%	3.6
VE3	19.2 ± 4.6%	3.8	22.0 ± 1.8%	4.4

#### Short Creep Tests

Short creep tests were conducted on both materials and for all the curing protocols outlined in Table 2, utilizing the same equipment described in Section 3.1. During these tests, the load was kept constant for a duration of 24 hours and the strain was measured using a Digital Image Correlation (DIC) system. The DIC system captured images at a frequency of 2 Hz throughout the loading phase. Following load application, images were recorded every 1 second for the initial 100 seconds, and subsequently at a rate of 2 images per minute.

#### Long Creep Tests

**Fig. 3:**
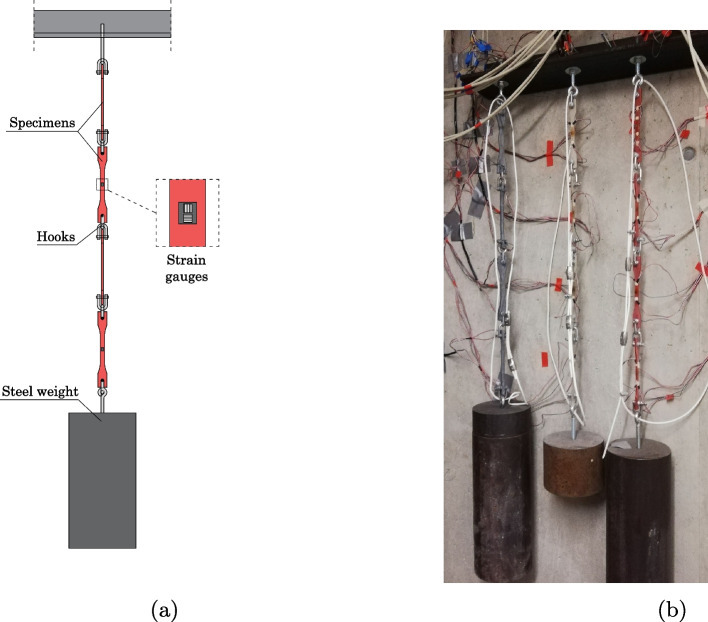
Set-up of tensile creep tests with dead load

Long-term tensile creep tests were executed on fully-cured epoxy samples (EP3) with the set-up shown in Fig. 3. Four "dog-bone" specimens were placed in series and connected by steel hooks. The chain of specimens was fixed from the top to a steel beam, while on the bottom end a steel weight was applied manually. The setup aims to investigate the secondary creep of the material during a long test duration. The load applied to the specimens is relatively low (20% of the maximum resistance) to ensure that it remains within the linear creep range and does not reach failure. Each dog-bone was equipped with four strain gauges (two 90^∘^ rosettes, Micro Measurements VPG) with an overall length of 4.17 mm and 120 Ohm resistance. The two rosettes were placed on each side and paired according to their orientation to form a full-bridge. In order to minimize the impact of sensors on the measurements, a protective coat and a thin layer of adhesive specifically designed for long term room temperature applications were applied. The data were acquired with a frequency of 1 Hz for the first 10 min and then decreased to 0.05 Hz. The tests were performed in a room with controlled temperature and humidity (21.5 ^∘^C / 55%) and lasted for approximately 2 years (744 days).

## Modeling of the Mechanical Response

**Fig. 4:**
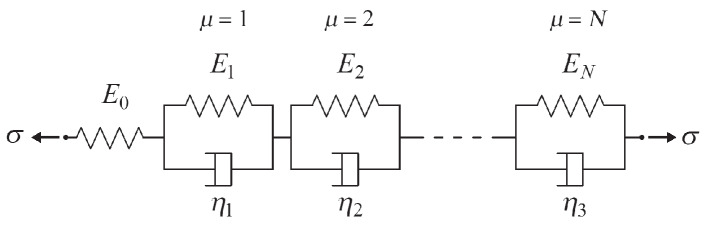
Schematic representation of the Kelvin chain

A simple analogy between force to stress and stretching to strain is used in rheological models to introduce a one-dimensional model through linear springs and dashpots. One of the most used is the Kelvin chain model as drawn in Fig. 4 [50]. It consists of a finite number of Kelvin units coupled in series, in which the first unit is typically a simple spring while the others are formed by a spring and a dashpot placed in parallel. This series models stress and stress rate depending on the strain and strain rates. As an example, we use $N=1$ that yields the so-called Zener model,

3 $$\begin{aligned} \sigma + K_1 \sigma ^\bullet = K_2 \varepsilon + K_3 \varepsilon ^\bullet \ , \end{aligned}$$

where this one-dimensional form may hold for the three-dimensional case in the same manner. This property is often called co-linearity. The material parameters, $K_1$, $K_2$, $K_3$ are in a direct relation with the chosen rheological model:

4 $$\begin{aligned} K_1 = \frac{\eta _1}{E_0 + E_1} \ , \quad K_2 = \frac{E_0 E_1}{E_0 + E_1} \ , \quad K_3 = \frac{E_0 \eta _1}{E_0 + E_1} \ . \end{aligned}$$

Obviously, by using $N=1$, only the first rate is incorporated. The stress or strain rate is (objective) time derivative and if *N* units are used, *N*-th rates in the material model can be obtained. In this way, the history of mechanical deformation is implemented into the model. This derivative causes its relaxation or retardation time. This quantity is given by the ratio of dashpot constant, viscosity $\eta _\mu$ and spring constant, representing Young’s modulus $E_\mu$. In the case of constant stress over time, such as in case of a typical sustained load / creep test, it is possible to solve this differential equation and obtain $\varepsilon = J \sigma$, where $\sigma$ is the constant loading and the so-called creep compliance, *J*, is the solution in time given as follows:

5 $$\begin{aligned} J = \frac{1}{E_0} + \sum _{\mu =1}^{N} \frac{1}{E_{\mu }} \bigg ( 1-\exp \Big (-\frac{t}{\tau _\mu } \Big ) \bigg ) \ . \end{aligned}$$

This solution belongs to the generalized Kelvin model and *N* is a parameter we need to choose. The parameters $E_0$ and $E_\mu$ are determined by setting the retardation times $\tau _\mu$ and using an inverse analysis based on an optimization algorithm that minimizes the squared error between the solution obtained from the parameters and the experimental results. The trust region reflective algorithm augmented by a sigmoid loss is employed for minimization, as implemented in SciPy in Python. The algorithm uses upper and lower bounds for parameters, hence, its reliability is an important asset providing a robust inverse identification method by choosing realistic bounds. The nonlinear optimization algorithm delivers results as compiled in Tables 5, 6, 7 and 8. The $\tau _\mu$ values were chosen in intervals $\Delta (\log \tau _{\mu }) = \log (10) = 1$ as in [59] to obtain a sufficiently smooth creep curve. The outcome of this fit is a series of parameters $E_{\mu }$. The plot of the compliance $1/E_{\mu }$ versus the logarithmic retardation time $\tau _{\mu }$ is called retardation spectrum of the material [62] and for a Kelvin chain model with a finite number of elements, this spectrum is discrete.

**Table 5:** Kelvin chain moduli fitted for tensile creep data

Product	$E_{0}$	$E_{1}$	$E_{2}$	$E_{3}$	Unit
EP1	$6.828\times 10^3$	$3.517\times 10^6$	$7.956\times 10^4$	$1.345\times 10^4$	[MPa]
EP2	$6.317\times 10^3$	$1.574\times 10^5$	$1.317\times 10^5$	$3.369\times 10^4$	[MPa]
EP3	$5.904\times 10^3$	$1.593\times 10^5$	$1.563\times 10^5$	$2.895\times 10^4$	[MPa]
VE1	$5.778\times 10^3$	$2.428\times 10^4$	$2.337\times 10^4$	-	[MPa]
VE2	$7.727\times 10^3$	$1.014\times 10^5$	$3.685\times 10^4$	$2.622\times 10^4$	[MPa]
VE3	$8.659\times 10^3$	$5.877\times 10^4$	$4.679\times 10^4$	$3.787\times 10^4$	[MPa]
$\tau _{\mu }$	-	0.01	0.1	1	[days]

**Table 6:** Kelvin chain moduli fitted for shear creep data

Product	$E_{0}$	$E_{1}$	$E_{2}$	$E_{3}$	Unit
EP1	$2.666\times 10^3$	$4.463\times 10^4$	$3.719\times 10^4$	$5.631\times 10^3$	[MPa]
EP2	$2.749\times 10^3$	$1.251\times 10^5$	$3.497\times 10^4$	$1.044\times 10^4$	[MPa]
EP3	$2.451\times 10^3$	$1.211\times 10^5$	$3.597\times 10^4$	$1.896\times 10^4$	[MPa]
VE1	$2.014\times 10^3$	$8.080\times 10^3$	$7.568\times 10^3$	$3.176\times 10^3$	[MPa]
VE2	$2.088\times 10^3$	$3.884\times 10^5$	$5.283\times 10^3$	-	[MPa]
VE3	$3.146\times 10^3$	$2.096\times 10^4$	$1.969\times 10^4$	$5.675\times 10^3$	[MPa]
$\tau _{\mu }$	-	0.01	0.1	1	[days]

**Table 7:** Spectra parameters fitted for tensile creep data

Product	$E_{0}$	$L(\tau )$		$C(\tau )$	
		*n*	$q_{2}$	*a*	*b*
EP1	$6.841\times 10^3$	0.710	$5.29\times 10^{-5}$	$2.07\times 10^{-5}$	0.660
EP2	$6.307\times 10^3$	0.367	$4.16\times 10^{-5}$	$8.76\times 10^{-6}$	0.293
EP3	$5.877\times 10^3$	0.264	$5.94\times 10^{-5}$	$1.06\times 10^{-5}$	0.334
VE1	$5.913\times 10^3$	0.363	$2.15\times 10^{-4}$	$5.36\times 10^{-5}$	0.341
VE2	$7.759\times 10^3$	0.289	$9.50\times 10^{-5}$	$1.49\times 10^{-5}$	0.204
VE3	$8.649\times 10^3$	0.093	$1.84\times 10^{-4}$	$8.77\times 10^{-6}$	0.054

**Table 8:** Spectra parameters fitted for shear creep data

Product	$E_{0}$	$L(\tau )$		$C(\tau )$	
		*n*	$q_{2}$	*a*	*b*
EP1	$2.670\times 10^3$	0.516	$1.68\times 10^{-4}$	$4.98\times 10^{-5}$	0.454
EP2	$2.749\times 10^3$	0.478	$1.04\times 10^{-4}$	$2.89\times 10^{-5}$	0.414
EP3	$2.463\times 10^3$	0.317	$8.84\times 10^{-5}$	$1.60\times 10^{-5}$	0.254
VE1	$2.074\times 10^3$	0.322	$6.30\times 10^{-4}$	$1.15\times 10^{-4}$	0.254
VE2	$2.123\times 10^3$	0.554	$3.61\times 10^{-4}$	$1.21\times 10^{-4}$	0.518
VE3	$3.238\times 10^3$	0.321	$2.46\times 10^{-4}$	$4.48\times 10^{-5}$	0.253

**Fig. 5:**
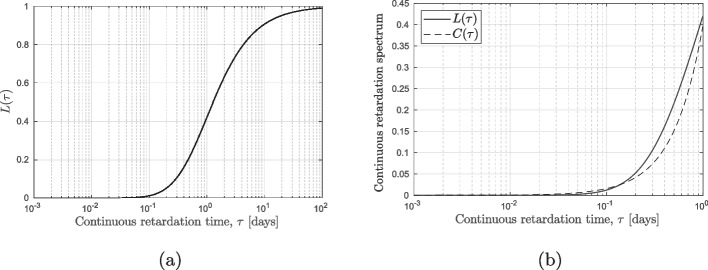
**(a)** Continuous retardation spectrum $L(\tau )$ for $n=1$ and $q_2 = 0.5$ from $10^{-3}$ to $10^{2}$ day and (**b)** Continuous retardation spectra $L(\tau )$ and $C(\tau )$ from $10^{-3}$ to 1 day

For creep tests with long loading times, a large number of elements in the chain is needed which would make the inverse identification procedure ill-posed and predictions error-prone. Instead of considering the many moduli in the chain as independent variables, they can be derived from a continuous (fit) function. The well-known *retardation spectrum* [62–68] can be helpful to increase the quality of the fit and improve the computational efficiency. For concrete, several continuous retardation spectra are found in the literature [62–70]. We employ the following form as an approximation to Eq. 5,

6 $$\begin{aligned} J(t)= \frac{1}{E_0} + \sum _{\mu =1}^{N} A_{\mu } \bigg ( 1-\exp \Big (-\frac{t}{\tau _\mu } \Big ) \bigg ) \ , \quad A_{\mu } = L(\tau ) \ln (10) \log (\tau _{\mu }) \ , \end{aligned}$$

in which $L(\tau )$ is the continuous retardation spectrum. By exploiting the Post–Widder formula [71, 72], it is possible to obtain

7 $$\begin{aligned} L(\tau ) = \lim _{k \rightarrow -\infty } {\frac{(-k\tau )^k}{(k-1)!}f^{(k)}(k\tau )} \ , \end{aligned}$$

where *k* is the desired order of approximation and $f^{(k)}$ is the *k*-th derivative of the compliance function. Considering the log-power compliance function:

8 $$\begin{aligned} J(t) = q_2 \ln \bigg ( 1+\Big (\frac{t}{\lambda _0}\Big )^n \bigg ) \ , \end{aligned}$$

it is possible to apply Eq. (4). Considering, $\lambda _0 = 1$ day and $k = 3$, the continuous retardation spectrum reads

9 $$\begin{aligned} \begin{aligned} L(\tau )=&\ \frac{-2n^2(3\tau )^{2n-3} \big ( n-1-(3\tau )^n \big ) }{ \big ( 1+(3\tau )^n \big )^3 } \frac{(3\tau )^2}{2} q_2 \\&+ \frac{n(n-2)(3\tau )^{(n-3)}\big ( n-1-(3\tau )^n \big ) - n^2(3\tau )^{2n-3}}{ \big ( 1+(3\tau )^n\big )^2} \frac{(3\tau )^2}{2}q_2 \end{aligned} \end{aligned}$$

in which, $q_2$ and *n* are the two parameters to-be-determined by experiments. We show in Fig. 5 the shape of the retardation function $L(\tau )$, considering $n=1$ and $q_2 = 0.5$ as an example. For short time ranges, it is possible to notice that the continuous retardation spectrum $L(\tau )$ is similar to a simple power law function (Fig. 5b). Since the time covered by the creep tests is relatively short, a simple retardation spectrum $C(\tau )$ described by the power law function is investigated as well,

10 $$\begin{aligned} C(\tau )=a\, \tau ^{b} \ , \end{aligned}$$

In which *a* and *b* are two terms to be fitted and $\tau$ is the continuous retardation time in days.

It is important to note that the generalized Kelvin model requires two assumptions: (i) linear viscoelasticity and (ii) a non-aging material. The first assumption is considered reasonable given the low load level applied to the materials. This implies that the materials can be assumed to exhibit linear elasticity, and no damage is expected to occur under such conditions. The two structural adhesives presented in this work have a thermoset polymer matrix; therefore, we consider them non-aging materials after acquiring the fully cured state (i.e., in protocols EP3 and VE3). For the other protocols, the short time range (24 h) of the creep tests is sufficiently short to neglect aging effects. Hence, we extend this assumption to the other curing states for the duration of the tests as well.

## Results

### Quasi-Static Tests

**Fig. 6:**
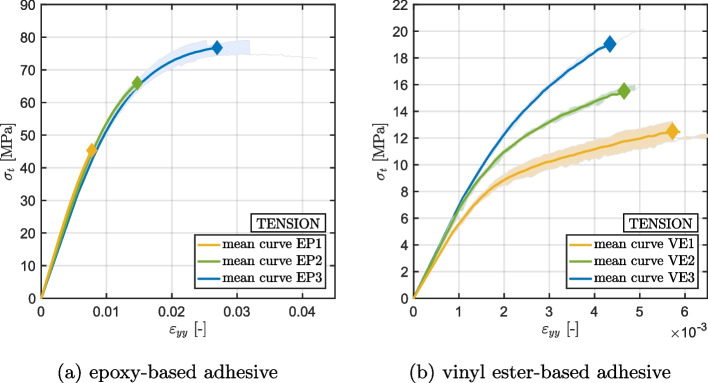
Results of the tensile tests performed on epoxy-based (**a**) and vinyl ester-based (**b**) adhesives. The mean value of four specimen results is shown as a continuous line, while the dispersion of all measurements has been indicated by the respective colored area

**Fig. 7:**
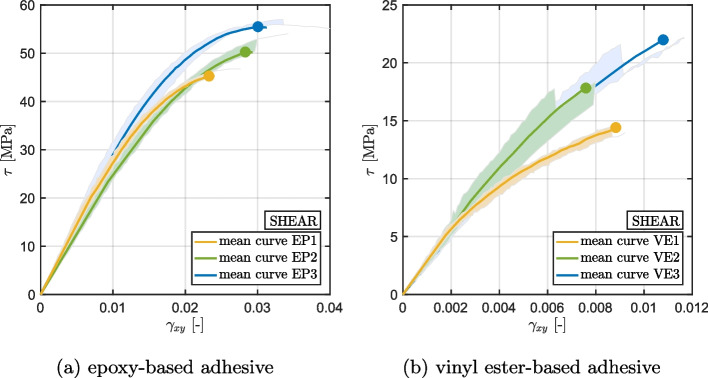
Results of the shear (Iosipescu) tests performed on epoxy-based (**a**) and vinyl ester-based (**b**) adhesives. The mean value of four specimen results is shown as a continuous line, while the dispersion of all measurements has been indicated by the respective colored area

Figures 6 and 7 present the stress-strain curves obtained respectively from the tensile and shear quasi-static tests performed on specimens with different curing and post-curing treatment. The outcome of the tensile and shear tests indicate that the extent of cure significantly affects the ultimate strengths of the materials. The results demonstrate that an increase in the degree of cure leads to a 69% increase in tensile strength and a 23% increase in shear strength of the epoxy-based material. Similarly, the vinyl ester based material exhibits a 54% increase in tensile strength and a 53% increase in shear strength upon an increase in the degree of cure. The behavior of the epoxy-based material in terms of stress-strain diagram remains unchanged with varying degrees of cure. In contrast, the vinyl ester based material demonstrates more ductile behavior for low-cured specimens while higher curing levels show a more brittle behavior. A detailed discussion of rate- and during-dependent effects on strength can be found in [34]. The recorded ultimate tensile strengths are reported in Table 4.

### Creep Tests

**Fig. 8:**
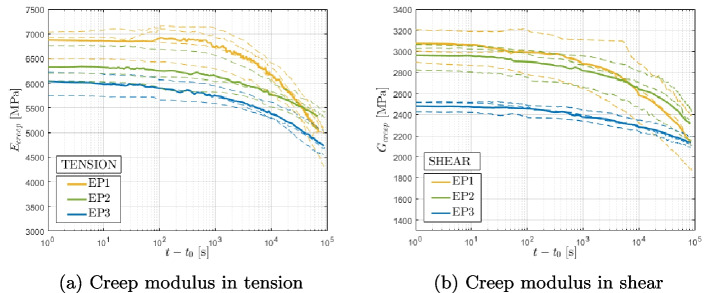
Epoxy-based adhesive, creep moduli in tension (**a**) and shear (**b**)

**Fig. 9:**
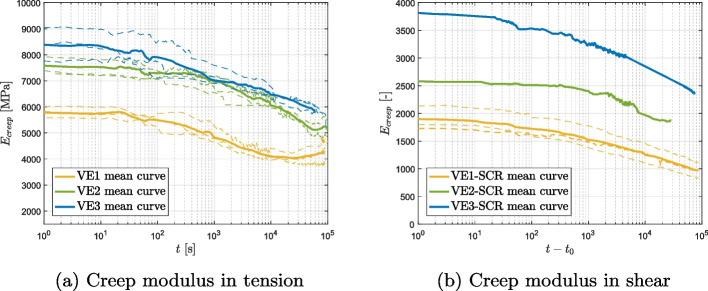
Vinyl ester-based adhesive, creep moduli in tension (**a**) and shear (**b**)

The creep moduli are depicted over time at different curing states in tension (a) and shear (b) in Figs. 8 and 9. The creep moduli, $E_\text {creep}$ and $G_\text {creep}$, are defined as the ratio between the sustained load applied to the specimen ($\sigma _\text {creep}$, $\tau _\text {creep}$) and the measured strain ($\epsilon _{yy}$, $\gamma _{xy}$). The complete strain versus logarithmic time is shown in Figs. 12 and 13. The results of the creep tests demonstrate a correlation between the degree of cure and both short-term and long-term behaviors of the two materials. Specimens with lower degrees of cure exhibit a more pronounced creep behavior compared to those with higher degrees of cure.

For the epoxy-based material, the creep behavior is consistent in both tension and shear loading conditions. Similarly, under both tensile and shear loads, the EP1 specimens exhibit a pronounced creep behavior, while the creep behaviors of EP2 and EP3 specimens are flatter and nearly identical under tensile loads. The tensile creep behavior of the vinyl ester is comparable to the one observed in the epoxy, while the shear curves do not reveal a clear trend. The quality of the vinyl ester data is unfortunately influenced by the presence of large fillers in the vinyl ester that can result in imperfections or micro-cracks at the microscopic level, introducing disturbances in the strain measurements obtained using DIC.

By comparing the two material, it is possible to notice that in the epoxy data, the initial modulus is similar for all curing conditions, with low-cured specimens exhibiting slightly higher initial stiffness compared to fully cured ones, while for vinyl ester data, the opposite trend is observed, with a higher difference between the initial modulus of VE1 and VE3. This phenomenon has been observed previously in [29, 31, 34].

**Fig. 10:**
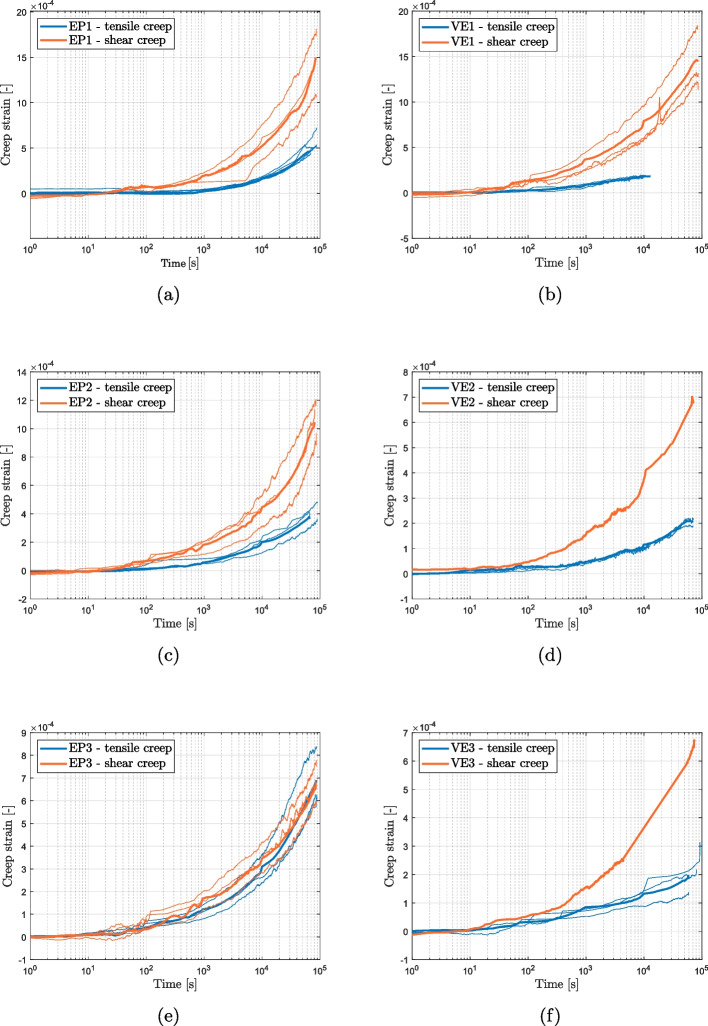
Comparison between shear and tensile creep

In Fig. 10, a comparison is shown between the creep strain in tension and shear for both materials, where creep strain is defined as the deformation $\varepsilon$ minus the initial strain $\varepsilon _0$. Notably, the difference between the two curves is more pronounced for EP1, the epoxy-based adhesive, and decreases for the fully cured (EP3) case. In contrast, for the vinyl ester, the difference in creep strain between tension and shear is consistent across all three cases.

**Fig. 11:**
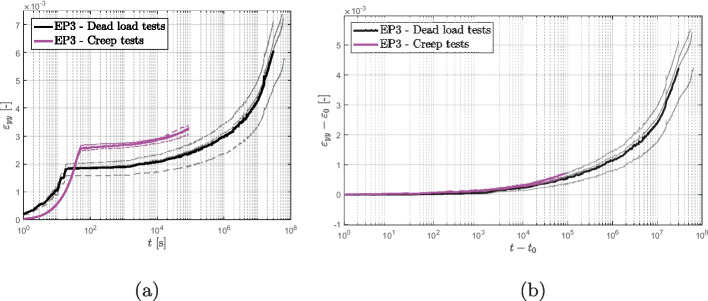
Comparison between short and long term measurements on epoxy EP3

The comparison of the short and long creep tests performed on the epoxy-based product is shown in Fig. 11. As previously discussed, the two experiments were conducted using different setups. The short creep tests were conducted using the ZwickRoell universal testing machine (UTM), where a constant load rate of 0.5 mm/min was applied until the target load was reached before switching to load control, resulting in a loading time of approximately 50 s. On the other hand, the long-term creep tests were carried out using the setup shown in Fig. 3, and the loads were applied manually over a time range of 20 s. This difference in the loading mode is reflected in a different initial strain, as seen in Fig. 11a. However, subtracting the initial strain reveals that the short and long term tests exhibit an identical trend (Fig. 11b), suggesting that the mode of control may not impact the long-term compliance.

### Modeling

The scope of this section is to show a comparison between the discrete Kelvin chain spectrum, the continuous retardation spectrum $L(\tau )$ as in Eq. (9), and the spectrum $C(\tau )$ as in Eq. (10). The determined parameters per each spectra are reported in Tables 5, 6, 7, and 8, and they are acquired via the inverse analysis.

**Fig. 12:**
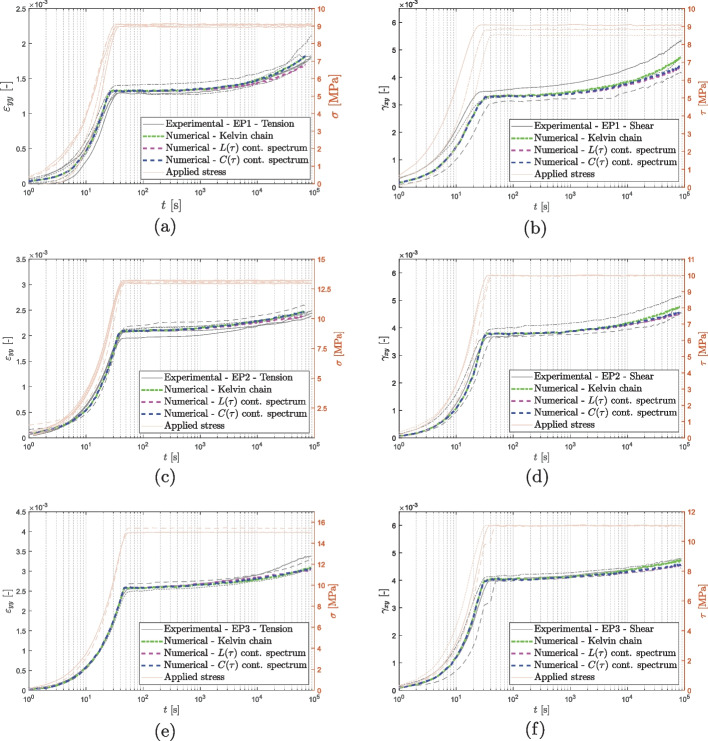
Epoxy: comparison between the experimental results and the modelings

**Fig. 13:**
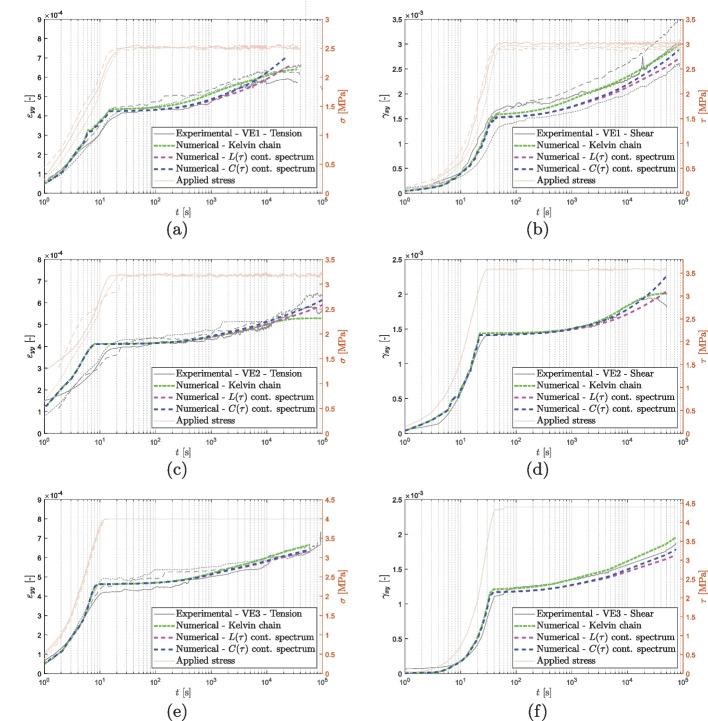
Vinyl ester: comparison between the experimental results and the modelings

Figures 12 and 13 compare the experimental data obtained from the short creep tests to the fit obtained applying the three spectra mentioned above. The curves obtained from the models are in close agreement with the experimental results, indicating that each of the spectra may be used to accurately predict the creep behavior of the material in the observed time period. Upon close examination of the results of the vinyl ester tests (as shown in Fig. 13a and d), it becomes evident that the discrete Kelvin chain spectrum is more susceptible to the quality of the data. The data for VE1 under tension and VE2 under shear presents some quality concerns, in the time range from $10^4$ to $10^5$ (corresponding to a retardation time of 1 day). In contrast, the continuous retardation spectra $L(\tau )$ and $C(\tau )$ are capable of compensating for these quality issues and provide a more stable prediction unlike the discrete spectrum, which closely follows the experimental curves including its fluctuations.

**Fig. 14:**
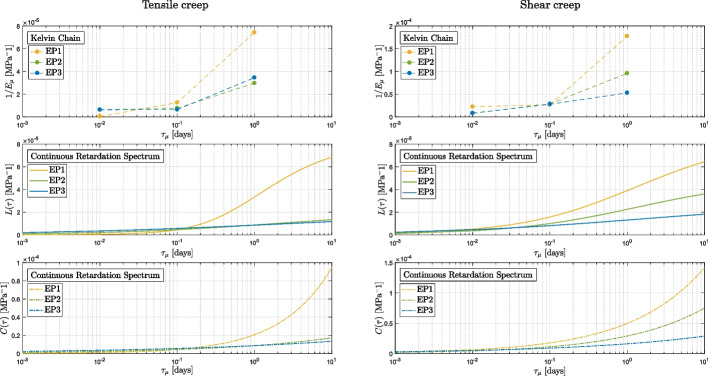
Tensile (left) and shear (right) spectra for the epoxy-based material. From top to bottom: discrete Kelvin Chain, continuous retardation spectrum $L(\tau )$ and continuous spectrum $C(\tau )$ based on the power law function

**Fig. 15:**
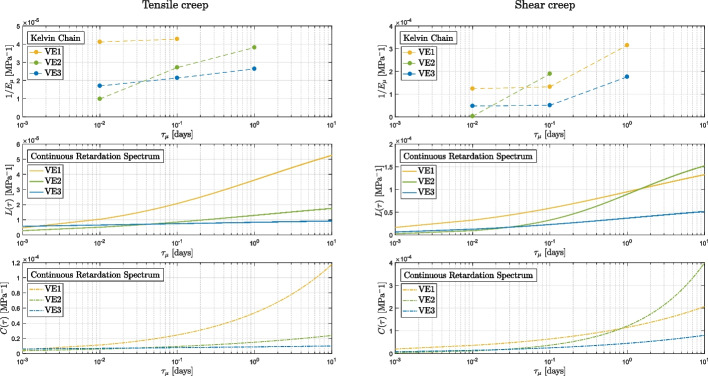
Tensile (left) and shear (right) spectra for the vinyl ester-based material. From top to bottom: discrete Kelvin Chain, continuous retardation spectrum $L(\tau )$ and continuous spectrum $C(\tau )$ based on the power law function

The difference in the creep behavior between the two materials at varying degrees of cure is not only reflected in the experimental curves but also in the shape of the spectra (Figs. 14 and 15). The curves for EP1 and VE1 in all three spectra exhibit a greater curvature compared to the other curves, indicating a higher creep of the material at lower curing states. It is noteworthy that the three spectra similarly reflect the impact of the degree of cure on the material’s behavior.

**Fig. 16:**
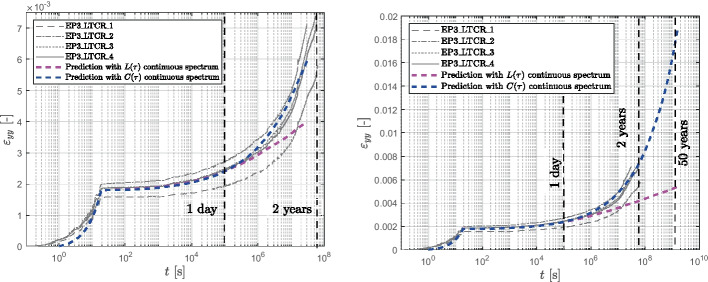
Predictions using the two continuous retardation spectrum for 2 years (**left**) and 50 years (**right**).

As previously discussed, the utilization of a continuous spectrum offers not only the advantage of a more robust inverse parameter identification (calibration) but is also expected to provide better extrapolations beyond the time scale covered by measurements. In order to verify this hypothesis, the two continuous retardation spectra were used to predict the available long-term creep data (EP3 with dead loads covering roughly 2 years), using the parameters $n, q_2, a$, and *b* obtained from the short-term tests of only 1 day. This extrapolation by almost three decades is challenging and the outcome is presented in logarithmic time in Fig. 16. The continuous retardation spectrum $C(\tau )$, based on the power law function, effectively predicts the long-term behavior of the epoxy, demonstrating that an accurate approximation of the behavior up to two years is achieved by extrapolating data obtained from a 1-day test. Conversely, the extrapolation produced by the continuous retardation spectrum $L(\tau )$, underestimates the creep behavior of the material.

**Fig. 17:**
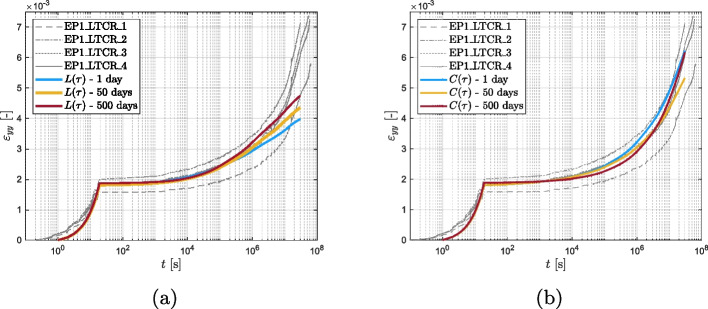
Two years prediction using continuous retardation spectrum $L(\tau )$ (**a**) and continuous retardation spectrum $C(\tau )$ (**b**), fitted on different time ranges: 1 day, 50 days and 500 days

For investigating the quality of the extrapolation, the parameters were fitted on different time ranges of the data: 1 day, 50 days, and 500 days. The outcomes of these fits are documented in Table 9 and shown in Fig. 17. Both Fig. 17a and b show that obviously a longer measurement based improves the predictions of the long-term data. However, the predictions based on the $L(\tau )$ spectrum still fall short while the $C(\tau )$ spectrum offers consistently adequate predictions.

**Table 9:** Spectra parameters fitted for shear creep data

Time range	$E_{0}$	$L(\tau )$		$C(\tau )$	
		*n*	$q_{2}$	*a*	*b*
1 day	$8.343\times 10^3$	0.264	$5.94\times 10^{-5}$	$1.06\times 10^{-5}$	0.334
50 days	$8.343\times 10^3$	0.524	$3.69\times 10^{-4}$	$9.56\times 10^{-5}$	0.303
500 days	$8.343\times 10^3$	0.630	$3.21\times 10^{-5}$	$6.70\times 10^{-5}$	0.427

#### Cure-Dependent Spectrum

**Fig. 18:**
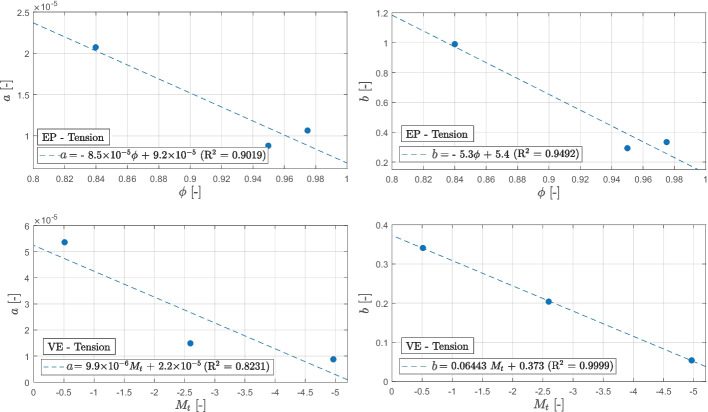
Linear relation between the two fitted parameters (*a* and *b*) and the degree of cure $\phi$ for the epoxy-based material and the mass change $M_t$ for the vinyl ester-based product in tension

**Fig. 19:**
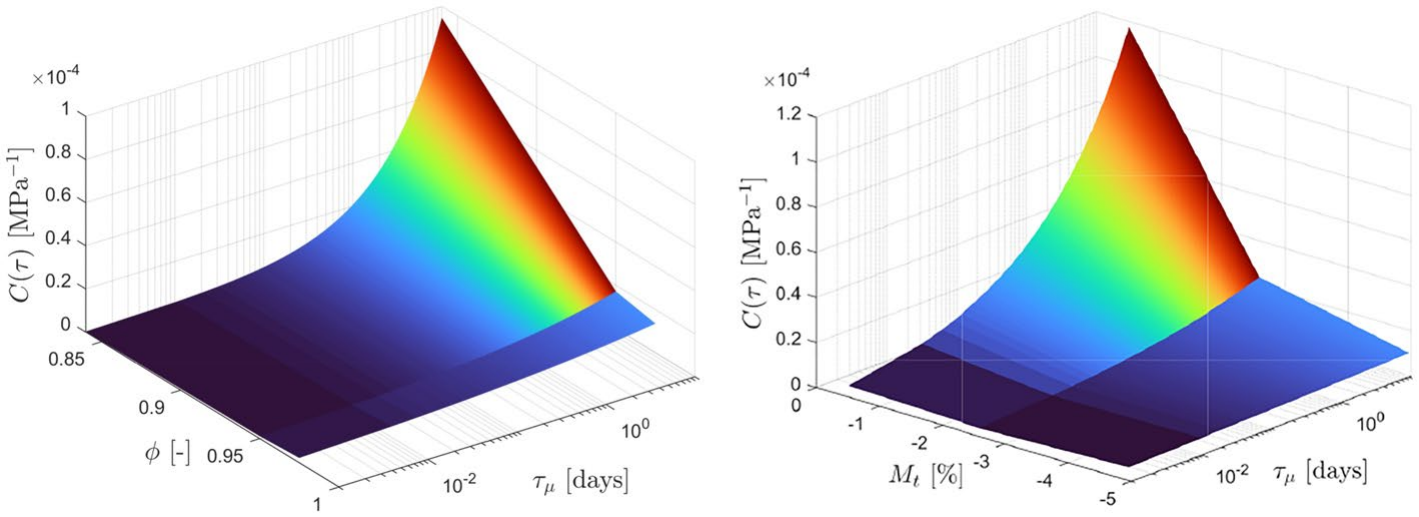
The $C(\tau )$ spectrum evolution for various degree of cure (EP on the left) and mass change (VE on the right)

The proposed spectra provide a means to characterize the creep behavior of the two materials by fitting two parameters. These parameters are influenced by the curing state of the thermoset, allowing for the establishment of a relationship between the two. Considering the fact that spectrum $C(\tau )$ is simpler than spectrum $L(\tau )$ and has been found to better describe the creep behavior, it will be utilized in this section for further analysis and discussion. Figures 18 and 19 shows the variations of the parameters *a* and *b* in relation to the degree of cure ($\phi$) for the epoxy-based adhesive and the mass change ($M_t$) for the vinyl ester-based material. As mentioned earlier in Section 2 and also discussed in the study by Daissé et al. [34], the determination of the degree of cure for the vinyl ester material did not provide conclusive findings. Hence, the mass change resulting from different curing and post-curing protocols is employed as an alternative measure. The relation between the two parameters and the curing states of the two materials can be described by a linear function. Table 10 presents a summary of the fitted equations and parameters for both materials. It is essential to emphasize that the quality of this fit is highly reliant on the quality of the acquired data and the number of evaluated curing states. However, despite these limitations, this approximation can serve as a foundational framework for a unified model that integrates creep behavior and the effects of curing.

**Table 10:** Linear relationship between the two fitted parameters *a* and *b* and the degree of cure $\phi$ for the epoxy-based product and mass change $M_t$ for the vinyl ester-based material

Product	Equation	R^2^
EP - Tension	$a = -8.5 \times 10^{-5} \phi + 9.17\times 10^{-5}$	0.9019
	$b = -5.305 \phi + 5.429$	0.9492
VE - Tension	$a = 9.898\times 10^{-6} M_t + 5.239\times 10^{-5}$	0.8231
	$b = 0.06443 M_t + 0.373$	0.9999
EP - Shear	$a = -0.0002 \phi + 0.0002$	0.9534
	$b = -1.1438 \phi + 1.429$	0.6025
VE - Shear	$a = 2\times 10^{-5} M_t + 0.0001$	0.7201
	$b = 0.0002 M_t + 0.2541$	-

## Conclusions

This contribution investigates the tensile and shear creep behavior of two, off-the-shelf, structural adhesives. The two materials were cured and post-cured with three different temperatures to imitate different in-situ conditions and then tested to study their short (1 day) and long-term (2 years) creep behavior. Valuable data for the research field are provided by this comprehensive experimental campaign. The results obtained from the creep tests were used to validate the generalized Kelvin model. Two continuous spectra were implemented and the results indicate a successful prediction of the experimental data. The two spectra are proposed as means to predict the long-term behavior of the adhesives using only short-term data, thereby contributing relevant knowledge for the assessment of life-time prediction. According to the results of the proposed study, the following conclusions are drawn:

- It exists a correlation between the degree of cure and the mechanical properties of epoxy and vinyl ester materials, including their ultimate tensile and shear strengths as well as their creep behavior. The results show that an increase in the degree of cure leads to an improvement in the mechanical properties of both materials, with a more pronounced effect on the vinyl ester material.
- The creep tests demonstrate that specimens with lower degrees of cure exhibit a more pronounced creep behavior compared to those with higher degrees of cure. This phenomenon is visible both in tension and in shear.
- The comparison of the short-term and long-term creep tests on the fully cured epoxy (EP3) indicates that the mode of control and speed of load application may not impact the long-term compliance.
- The generalized Kelvin model is suitable to correctly replicate the behavior of the two materials both in tension and in shear in the domain of linear viscoelasticity. However, additional investigations will be needed to confirm the modelling assumptions as well as the influence of the stress level.
- The discrete spectrum and the two continuous retardation spectra lead to adequate results. The comparison of the three spectra shows that each of them may be used to accurately predict the creep behavior of the material, with the continuous retardation spectra being more robust in presence of experimental noise.
- The difference between tensile and shear creep strain is significant. For the epoxy-based adhesive, it is more evident in the EP1 and decreases for the fully cured (EP3) case, while for the vinyl ester, the difference in creep strain between tension and shear is consistent across all three reference states.
- Both continuous spectra can be used to extrapolate the creep behavior in time. However, the investigated continuous spectrum described by the power law function offers superior predictions for the investigated material. The parameter fitted on 1-day creep tests adequately predict the 2 years experiments.
- The proposed model will serve as a fundamental tool for accurately modeling the behavior of the two materials under low stress conditions. To provide a reliable prediction of the lifespan of the products, this model will need to be complemented by tertiary creep and damage models. This comprehensive model will enhance our understanding of the long-term performance and durability of the materials and facilitates a more accurate life predictions.

## Data Availability

The datasets generated and analysed during the current study are available from the corresponding author on reasonable request.

## References

[1] Feng C-W, Keong C-W, Hsueh Y-P, Wang Y-Y, Sue H-J (2005). Modeling of long-term creep behavior of structural epoxy adhesives. Int. J. Adhes. Adhes..

[2] ACI Committee 355: Qualification of Post-Installed Adhesive Anchors in Concrete (ACI 355.4M-11) and Commentary. American Concrete Institute (2017)

[3] ASTM E1512: Standard test methods for testing bond performance of adhesive bonded anchors. American Society for Testing and Materials (ASTM) (2001)

[4] EOTA - ETAG 001-5: Guideline for european technical approval of metal anchors for use in concrete - part five: Bonded anchors. European Organisation for Technical Approvals (2011)

[5] Boumakis, I., Ninčević, K., Vorel, J., Wan-Wendner, R.: Creep rate based time-to-failure prediction of adhesive anchor systems under sustained load. Compos. Part B Eng. **178**, 107389 (2019). 10.1016/j.compositesb.2019.107389

[6] Meissl, S., Ninčević, K., Abali, B., Wan-Wendner, R.: Mortar cure-dependent effects on adhesive anchor systems loaded in tension. Constr. Build. Mater. **362**, 129554 (2023). 10.1016/j.conbuildmat.2022.129554

[7] NTSB/HAR: Ceiling collapse in the interstate 90 connector tunnel. Tech. rep., Washington, D.C. (2006)

[8] Kawahara, S., Doi, H., Shirato, M., Kajifusa, N., Kutsukake, T.: Investigation of the tunnel ceiling collapse in the central expressway in Japan. TRB 93th Annual Meeting, Washington D.C. (2014)

[9] Shirato, M.: Investigation of tunnel ceiling collapse accident. 30th US - Japan Bridge Engineering Workshop At: Tsukuba, Japan (2014)

[10] Muciaccia, G., Consiglio, A., Rosati, G.: Creep behavior of bonded anchor under high sustained loading at long term temperature. In: Hordijk, D.A., Luković, M. (eds.) High Tech Concrete: Where Technology and Engineering Meet, pp. 956–964. Springer, Cham (2018)

[11] Mahadik V, Hofmann J (2019). Creep behaviour of tension loaded adhesive anchors in non-cracked low strength concrete. IOP Conf. Ser. Mater. Sci. Eng..

[12] El Menoufy, A.M.: Creep behaviour of post-installed adhesive anchors under various sustained load levels and environmental exposures (2010)

[13] Djeumen, E., Chataigner, S., Créac’hcadec, R., Sourisseau, Q., Quéméré, M.O., Court, J.P., Sayed, F.: Creep investigations on adhesively bonded fasteners developed for offshore steel structures. Mar. Struct. **69**, 102660 (2020). 10.1016/j.marstruc.2019.102660

[14] Yang L, Chen F, Yin H (2017). Creep and damage of an adhesive anchor system by accelerated testing and modeling. Int. J. Damage Mech..

[15] Yang, M., Zhao, Y., Zhang, N.: Creep behavior of epoxy-bonded anchor system. Int. J. Rock Mech. Min. Sci. **67**, 96–103 (2014). 10.1016/j.ijrmms.2014.02.001

[16] Meshgin P, Choi K-K, Reda Taha MM (2009). Experimental and analytical investigations of creep of epoxy adhesive at the concrete-frp interfaces. Int. J. Adhes. Adhes..

[17] Sadigh, M.A., Paygozar, B., Silva, L.F.M., Martínez Pañeda, E.: Creep behaviour and tensile response of adhesively bonded polyethylene joints: Single-lap and double-strap. Int. J. Adhes. Adhes. **102**, 102666 (2020). 10.1016/j.ijadhadh.2020.102666

[18] de Zeeuw, C., Teixeira de Freitas, S., Zarouchas, D., Schilling, M., Lopes Fernandes, R., Dolabella Portella, P., Niebergall, U.: Creep behaviour of steel bonded joints under hygrothermal conditions. Int. J. Adhes. Adhes. **91**, 54–63 (2019). 10.1016/j.ijadhadh.2019.03.002

[19] Carneiro Neto, R.M., Paula, M.S.d., Sampaio, E.M., Rohem, N.R.F., Vignoli, L.L.: Study of the life span of adhesively bonded single lap joint due to creep. Matéria (Rio de Janeiro) **26**(4) (2021). 10.1590/S1517-707620210004.1321

[20] Zuo, P., Vassilopoulos, A.P.: Review of fatigue of bulk structural adhesives and thick adhesive joints. Int. Mater. Rev. **66**(5), 313–338 (2021). 10.1080/09506608.2020.1845110

[21] ISO 527-1:2012: Plastics - Determination of tensile properties, Part 1: General principles. International Organization for Standardization (2012)

[22] ASTM D5379: Standard Test Method for Shear Properties of Composite Materials by the V-Notched Beam Method1. American Society for Testing and Materials (ASTM) (1998)

[23] Ziaee S, Palmese GR (1999). Effects of temperature on cure kinetics and mechanical properties of vinyl-ester resins. J. Polym. Sci., Part B: Polym. Phys..

[24] Zhang X, Bitaraf V, Wei S, Guo Z, Zhang X, Wei S, Colorado HA (2014). Vinyl ester resin: Rheological behaviors, curing kinetics, thermomechanical, and tensile properties. AIChE J..

[25] Czaderski C, Martinelli E, Michels J, Motavalli M (2012). Effect of curing conditions on strength development in an epoxy resin for structural strengthening. Compos. B Eng..

[26] Abali, B.E., Vorel, J., Wan-Wendner, R.: Thermo-mechano-chemical modeling and computation of thermosetting polymers used in post-installed fastening systems in concrete structures. Contin. Mech. Thermodyn. 1–20 (2020) 10.1007/s00161-020-00939-4 PMC10154286 37152696

[27] Hossain M, Possart G, Steinmann P (2009). A small-strain model to simulate the curing of thermosets. Comput. Mech..

[28] Liao, Z., Yao, X., Zhang, L., Hossain, M., Wang, J., Zang, S.: Temperature and strain rate dependent large tensile deformation and tensile failure behavior of transparent polyurethane at intermediate strain rates. Int. J. Impact Eng. **129**, 152–167 (2019). 10.1016/j.ijimpeng.2019.03.005

[29] Singer, G., Sinn, G., Schwendtner, K., Lichtenegger, H., Wan-Wendner, R.: Time dependent changes of mechanical properties of polymer-based composite materials for adhesive anchor systems. Compos. Struct. **196** (2018). 10.1016/j.compstruct.2018.04.076

[30] Singer G, Sinn G, Lichtenegger HC, Veigel S, Zecchini M, Wan-Wendner R (2019). Evaluation of in-situ shrinkage and expansion properties of polymer composite materials for adhesive anchor systems by a novel approach based on digital image correlation. Polym. Test..

[31] Siedlaczek, P., Sinn, G., Peter, P., Wan-Wendner, R., Lichtenegger, H.C.: Characterization of moisture uptake and diffusion mechanisms in particle-filled composites. Polymer **249**, 124799 (2022). 10.1016/j.polymer.2022.124799

[32] Abali, B.E., Yardımcı, M.Y., Zecchini, M., Daissé, G., Marchesini, F.H., De Schutter, G., Wan-Wendner, R.: Experimental investigation for modeling the hardening of thermosetting polymers during curing. Polym. Test. **102**, 107310 (2021). 10.1016/j.polymertesting.2021.107310

[33] Abali, B.E., Zecchini, M., Daissé, G., Czabany, I., Gindl-Altmutter, W., Wan-Wendner, R.: Cure kinetics and inverse analysis of epoxy-amine based adhesive used for fastening systems. Materials **14**(14) (2021). 10.3390/ma14143853 10.3390/ma14143853 PMC8354013 34300770

[34] Daissé, G., Marcon, M., Zecchini, M., Wan-Wendner, R.: Cure-dependent loading rate effects on strength and stiffness of particle-reinforced thermoset polymers. Polymer **259**, 125326 (2022). 10.1016/j.polymer.2022.125326

[35] Lapique F, Redford K (2002). Curing effects on viscosity and mechanical properties of a commercial epoxy resin adhesive. Int. J. Adhes. Adhes..

[36] Dodiuk H, Kenig S (1994). Low temperature curing epoxies for structural repair. Prog. Polym. Sci..

[37] Matsui K (1990). Effects of curing conditions and test temperatures on the strength of adhesive-bonded joints. Int. J. Adhes. Adhes..

[38] Silva, P., Fernandes, P., Sena-Cruz, J., Xavier, J., Castro, F., Soares, D., Carneiro, V.: Effects of different environmental conditions on the mechanical characteristics of a structural epoxy. Compos. Part B Eng. **88**, 55–63 (2016). 10.1016/j.compositesb.2015.10.036

[39] Filanova, Y., Hauptmann, J., Längler, F., Naumenko, K.: Inelastic behavior of polyoxymethylene for wide strain rate and temperature ranges: Constitutive modeling and identification. Materials **14**(13) (2021). 10.3390/ma14133667 10.3390/ma14133667 PMC8269818 34279248

[40] Spathis G, Kontou E (2012). Creep failure time prediction of polymers and polymer composites. Compos. Sci. Technol..

[41] Carneiro Neto, R.M., Akhavan-Safar, A., Sampaio, E.M., Assis, J.T., da Silva, L.F.M.: Assessment of the creep life of adhesively bonded joints using the end notched flexure samples. Eng. Fail. Anal. **133**, 105969 (2022). 10.1016/j.engfailanal.2021.105969

[42] Kyzy, B.K., Lanzutti, A., Magnan, M., Rondinella, A., Simonato, M., Furlanetto, R., Fedrizzi, L.: Creep study of glass reinforced polypropylene: Effect of temperature and presence of notches. Eng. Fail. Anal. **128**, 105624 (2021). 10.1016/j.engfailanal.2021.105624

[43] Benzarti, K., Houhou, N., Quiertant, M., Chataigner, S.: Creep behavior of cold-curing epoxy adhesives: Analysis and predictive approach. Proceedings of the 7th International Conference on FRP Composites in Civil Engineering, CICE 2014 (2014)

[44] Faraz, I., Besseling, N., Korobko, A., Picken, S.J.: Characterization and modeling of creep behavior of a thermoset nanocomposite. Polym. Compos. **36** (2015). 10.1002/pc.22946

[45] Li, H., Luo, Y., Hu, D.: Long term creep assessment of room-temperature cured epoxy adhesive by time-stress superposition and fractional rheological model. Appl. Rheol. **28**(6), 201864796 (2018). 10.3933/applrheol-28-64796

[46] Majda P, Skrodzewicz J (2009). A modified creep model of epoxy adhesive at ambient temperature. Int. J. Adhes. Adhes..

[47] Costa I, Barros J (2015). Tensile creep of a structural epoxy adhesive: Experimental and analytical characterization. Int. J. Adhes. Adhes..

[48] Silva, P., Valente, T., Azenha, M., Sena-Cruz, J., Barros, J.: Viscoelastic response of an epoxy adhesive for construction since its early ages: Experiments and modelling. Compos. Part B Eng. **116**, 266–277 (2017). 10.1016/j.compositesb.2016.10.047

[49] Altenbach, H., Girchenko, A., Kutschke, A., Naumenko, K.: Inelastic behavior of materials and structures under monotonic and cyclic loading, 1–15 (2015). 10.1007/978-3-319-14660-7_1

[50] Naumenko, K., Altenbach, H.: Modeling of creep for structural analysis, 17–84 (2007). 10.1007/978-3-540-70839-1_2

[51] Dean GD, Broughton W (2007). A model for non-linear creep in polypropylene. Polym. Test..

[52] Dean, G.D., McCartney, L.N., Mera, R., Urquhart, J.M.: Modeling nonlinear viscoelasticity in polymers for design using finite element analysis. Polym. Eng. Sci. **51**(11), 2210–2219. 10.1002/pen.21993

[53] Nosrati, N., Zabett, A., Sahebian, S.: Stress dependency of creep response for glass/epoxy composite at nonlinear and linear viscoelastic behavior. Int. J. Polym. Sci. **2022** (2022). 10.1155/2022/9733138

[54] Fischer, J., R Bradler, P., W Lang, R., Wan-Wendner, R.: Long-term creep behavior of resin-based polymers in the construction industry. Mater. Today Commun. **18**, 60–65 (2019). 10.1016/j.mtcomm.2018.11.006

[55] R Bradler, P., Fischer, J., Wan-Wendner, R., W Lang, R.: Shear test equipment for testing various polymeric materials by using standardized multipurpose specimens with minor adaptions. Polym. Test. **75**, 93–98 (2019). 10.1016/j.polymertesting.2019.01.024

[56] Sullivan JL, Blais EJ, Houston D (1993). Physical aging in the creep behavior of thermosetting and thermoplastic composites. Compos. Sci. Technol..

[57] Dorléans, V., Delille, R., Notta-Cuvier, D., Lauro, F., Michau, E.: Time-temperature superposition in viscoelasticity and viscoplasticity for thermoplastics. Polym. Test. **101**, 107287 (2021). 10.1016/j.polymertesting.2021.107287

[58] Achereiner, F., Engelsing, K., Bastian, M., Heidemeyer, P.: Accelerated creep testing of polymers using the stepped isothermal method. Polym. Test. **32**, 447–454 (2013). 10.1016/j.polymertesting.2013.01.014

[59] Bažant, Z.P., Carol, I.: Fifth Rilem international symposium on creep and shrinkage of concrete (CONCREEP-5). Mater. Struct. **27** (1994). 10.1007/BF02473431

[60] Singer, G., Siedlaczek, P., Sinn, G., Kirner, P.H., Schuller, R., Wan-Wendner, R., Lichtenegger, H.C.: Vacuum casting and mechanical characterization of nanocomposites from epoxy and oxidized multi-walled carbon nanotubes. Molecules **24**(3) (2019). 10.3390/molecules24030510 10.3390/molecules24030510 PMC6384675 30708980

[61] Hossain, M., Steinmann, P.: Degree of cure-dependent modelling for polymer curing processes at small-strain. part i: consistent reformulation. Comput. Mech. **53**(4), 777–787 (2014). 10.1007/s00466-013-0929-5

[62] Bazant, Z., Jirásek, M.: Creep and hygrothermal effects in concrete structures. (2018). 10.1007/978-94-024-1138-6

[63] Bažant, Z., Baweja, S.: Creep and shrinkage prediction model for analysis and design of concrete structures: Model b3. ACI SpecialPublicationCreep and ShrinkageofConcrete (2000)

[64] Bažant Z, Wu S (1973). Dirichlet series creep function for aging concrete. J. Eng. Mech. - ASCE.

[65] Bažant, Z.: Viscoelasticity of solidifying porous material-concrete. CBI Forsk (5) (1977)

[66] Bažant, Z.P., Prasannan, S.: Solidification theory for concrete creep. i: formulation. J. Eng. Mech. - ASCE **115**, 1691–1703 (1989)

[67] Bažant, Z.P., Hauggaard, A.B., Baweja, S., Ulm, F.-J.: Microprestress-solidification theory for concrete creep. i: Aging and drying effects. J. Eng. Mech. - ASCE **123**, 1188–1194 (1997)

[68] Jirásek, M., Havlasek, P.: Accurate approximations of concrete creep compliance functions based on continuous retardation spectra. Computers I & Structures **135**, 155–168 (2014). 10.1016/j.compstruc.2014.01.024

[69] FIB: Model Code for Concrete Structures 2010. Fédération internationale de béton. Ernst & Sohn (2013)

[70] JSCE: Standard specifications for concrete structures - 2007 “design”. Japan Society of Civil Engineers Guidelines for Concrete, No. 15. (2007)

[71] Widder, D.V.: Laplace transform (PMS-6) **64** (2015)

[72] Widder, D.V.: An introduction to transform theory. **42** (1971)

